# Organic Cation Dynamics in the Layered Lead Iodide Perovskites BA_2_PbI_4_ and PEA_2_PbI_4_

**DOI:** 10.1021/acs.jpclett.5c02091

**Published:** 2025-09-26

**Authors:** Rasmus Lavén, Michael M. Koza, Niina H. Jalarvo, Marco Moroni, Lorenzo Malavasi, Maths Karlsson

**Affiliations:** † Department of Chemistry and Chemical Engineering, 11248 Chalmers University of Technology, Göteborg 41296, Sweden; ‡ Institut Laue-Langevin, 71 avenue des Martyrs, CS 20156, 38042 Grenoble cedex 9, France; § Chemical and Engineering Materials Division, 6146 Oak Ridge National Laboratory, Oak Ridge, Tennessee 37831-6475, United States; ∥ Department of Chemistry and INSTM, University of Pavia, Viale Taramelli 16, Pavia 27100, Italy

## Abstract

We report on a quasielastic neutron scattering (QENS) study of the rotational dynamics of organic cations in the optoelectronic layered metal halide perovskites BA_2_PbI_4_ (BA = butylammonium, CH_3_(CH_2_)_3_NH_3_) and PEA_2_PbI_4_ (PEA = phenethylammonium, C_6_H_5_(CH_2_)_2_NH_3_). For BA_2_PbI_4_, the measurements reveal highly temperature dependent dynamics. Between 100 and 260 K, the dynamics are ascribed to rotational dynamics of the -CH_3_ and -NH_3_ groups of the BA cation, whereas above 300 K we also observe the additional presence of full rotations of the whole BA cation around its long molecular axis. For PEA_2_PbI_4_, the measurements reveal dynamics that are more spatially restricted in nature, i.e., rotational dynamics of the -NH_3_ groups of the PEA cations for temperatures above 200 K, with no other dynamical processes observed at higher temperatures. A correlation of the dynamics results to literature data on the optical properties of the materials suggests that the more restricted organic cation dynamics in PEA_2_PbI_4_ are related to its longer lifetime and diffusion lengths for charge carriers, as compared to BA_2_PbI_4_.

## Graphical abstract

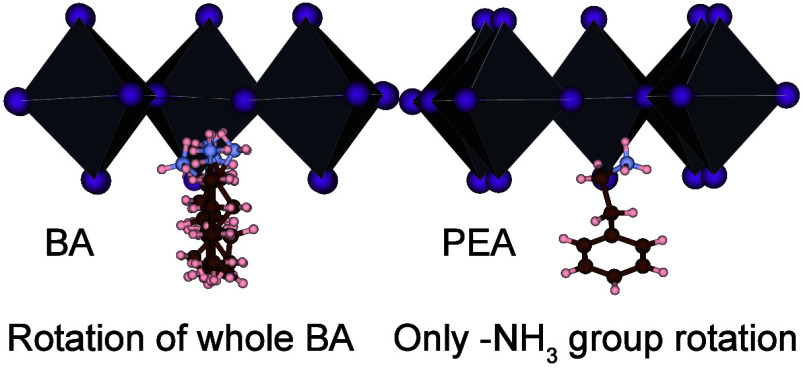

Layered, also known as two-dimensional (2D), metal halide perovskites (MHPs), such as Ruddlesden–Popper-type, R_2_BX_4_, structured materials, where R is an organic cation, B is a divalent metal cation, and X is a halide anion, and for which inorganic layers of corner-sharing BX_6_ octahedra are sandwiched between the organic cations, are currently receiving considerable attention. This is mainly because of their versatile and useful optoelectronic properties and concomitant promise for application in both solar cells^1,2^ and light-emitting diodes.^3,4^ Additionally, they show potential for application in low-dimensional magnetism^5^ and thermoelectrics.^6^ However, a long-standing challenge for MHPs generally is to understand how the optoelectronic properties are affected by the organic cation dynamics.

To date, most studies focusing on investigations of the organic cation dynamics in MHPs have been performed on nonlayered, three-dimensional (3D), materials, such as [CH_3_NH_3_]­PbX_3_ (X = I, Br, and Cl) and [HC­(NH_2_)_2_]­PbX_3_ (X = I and Br). The results from these studies have, typically, unraveled various rotational (reorientational) motions of the organic cations,^7–14^ which in some cases have been linked to the materials’ optoelectronic properties.^15^ For layered MHPs, the organic cation dynamics have been studied using nuclear magnetic resonance (NMR),^16–20^ QENS,^21–23^ and molecular dynamics (MD) simulations,^24,25^ but to a lesser extent than the 3D materials. Interestingly, a recent QENS study reported on a correlation between organic cation dynamics, especially the “dynamic cation radius”, and luminescence in the layered MHPs BA_2_PbBr_4_, ODAPbBr_4_, and GABA_2_PbBr_4_ (ODA = 1,8-diaminooctammonium, and GABA = 4-aminobutyric acid),^22^ thus strengthening the hypothesis that the organic cation dynamics play a prominent role in the optoelectronic properties of MHPs generally.

Here, in a variable-temperature QENS study, we investigate the nature of organic cation dynamics in the prototypical layered MHPs BA_2_PbI_4_ and PEA_2_PbI_4_.^26,27^ While BA_2_PbI_4_ and PEA_2_PbI_4_ exhibit the same inorganic substructure, they differ in that the BA cation consists of an alkyl chain with a terminal -NH_3_ group, whereas the PEA cation also includes a phenyl ring (Figure 1). In addition, while BA_2_PbI_4_ transforms from a low-temperature (LT) orthorhombic structure to an iso-symmetric high-temperature (HT) orthorhombic structure (both phases have space group *Pbca*) at around 240 K on cooling and 270 K on heating, PEA_2_PbI_4_ exhibits a triclinic structure (space group *P*1̅) up to 400 K.^28,29^ Interestingly, BA_2_PbI_4_ and PEA_2_PbI_4_ show distinct differences in their optoelectronic properties; PEA_2_PbI_4_ exhibits slower charge recombination and longer charge carrier lifetimes^30–32^ and faster and longer diffusion lengths of excitons compared to those of BA_2_PbI_4_.^33^ Therefore, the choice of materials for our study serves as an excellent model system for investigating the impact of organic cation dynamics on both phase transitions and optoelectronic properties in layered MHPs.

**Figure 1:**
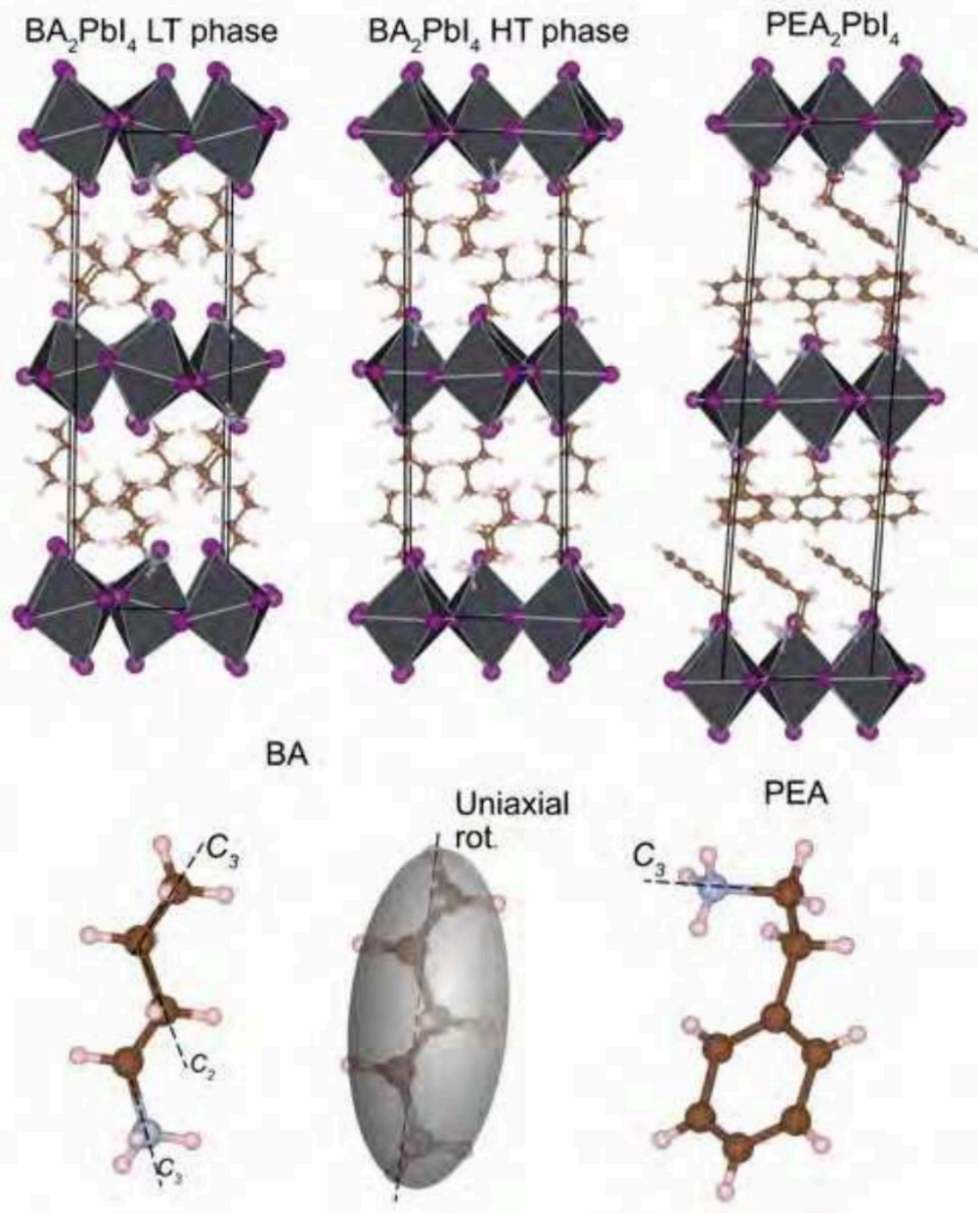
Schematic illustration (top) of the crystal structures of BA_2_PbI_4_ and PEA_2_PbI_4_.^28,34^ I atoms are illustrated as purple spheres, and Pb atoms lie within the black octahedra. Illustration of the BA and PEA cations (bottom), together with their possible dynamics. The shaded gray ellipsoid indicates the uniaxial rotation around the long molecular axis of BA_2_PbI_4_.^21^ Figures were made using VESTA.^35^

Panels a–c of Figure 2 show data from elastic fixed window scans (EFWSs) and inelastic fixed window scans (IFWSs) for both materials; for details of the experiments, see Experimental Details. First, we consider the data for BA_2_PbI_4_. As one can see in panels a and b of Figure 2, the EFWS of this material is characterized by a slight and essentially linear decrease in the elastic intensity from 20 to ∼100 K with an increase in temperature. This Debye–Waller-like behavior manifests the gradual activation of vibrational dynamics in the material. Upon further heating, the elastic intensity decreases at a higher rate, with a marked decrease at around 270 K. This decrease marks the onset of relaxation dynamics in the material. In comparison, we observe an essentially reversed behavior upon cooling (Figure 2c), except that the marked feature at 270 K is now shifted to around 240 K, thus indicating some hysteresis of the relaxational dynamics. Note, the marked features at 270 and 240 K in the elastic intensity are in good agreement with the reported phase transition temperatures upon heating (270 K) and cooling (250 K), respectively,^36^ which thus indicates different organic cation dynamics in the two crystal phases. Considering next the IFWS (Figure 2a–c), we observe that the inelastic intensity displays two maxima, at around 160 and 240 K, which point toward the presence of (at least) two different relaxational dynamic processes in the material.

**Figure 2:**
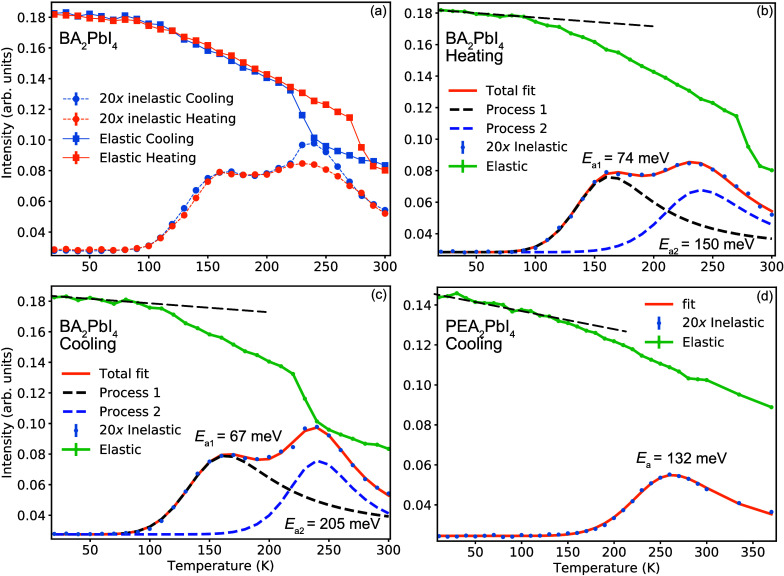
EFWSs and IFWSs for (a–c) BA_2_PbI_4_ and (d) PEA_2_PbI_4_, summed over all *q* values, as measured on BASIS with the Si(111) analyzer crystals. The black dashed lines are guides to the eyes to illustrate the essentially linear behavior from the Debye–Waller factor below ≈100 K for BA_2_PbI_4_ and below ≈150 K for PEA_2_PbI_4_. The inelastic intensities have been multiplied by a factor of 20 for increased visibility. Error bars are smaller than the size of the symbols.

Next, we consider the data for PEA_2_PbI_4_ (see Figure 2d). Like that of BA_2_PbI_4_, the EWFS of PEA_2_PbI_4_ shows a slight and virtually linear variation of the elastic intensity in the low-temperature range, here from 2 to ∼150 K, whereas the faster rate of the decrease in the elastic intensity at higher temperatures marks the onset of relaxational dynamics. However, different from that of BA_2_PbI_4_, the decrease in the elastic intensity up to 150 K is significantly larger, which indicates generally larger vibrational amplitudes. Furthermore, the elastic intensity does not show any additional marked features; rather, a quite smooth variation of the elastic intensity is observed from 150 to 300 K. This is in agreement both with the absence of phase transitions in this temperature range^28^ and with the observation of a single peak in the IFWS, which suggests that there is only one dynamical process in PEA_2_PbI_4_ on the time scale probed here.

For a quantitative analysis of the dynamics in both materials, the respective IFWS was fitted to the function *I*(ω, *T*) ∝ [τ­(*T*)/(1 + ω^2^τ­(*T*)^2^)] + *c*. This function describes the inelastic intensity at a particular energy transfer ω and temperature *T*, for a single relaxational process with a relaxation time τ,^37^ where *c* is a constant. The relaxation time is assumed to follow an Arrhenius law according to the equation τ­(*T*) = τ_0_ exp­(*E*_a_/*k*_B_ *T*), where *E*_a_ is the activation energy of the dynamics, τ_0_ is the trial frequency, and *k*_B_ is the Boltzmann constant.^37^ Note that, while at the lowest temperature (10 K) there is no dynamics and hence the scattering is fully elastic, as the temperature increases the dynamics become faster and, at a certain temperature, enter the slower time scale limit of the observation time window of the instrument. This gives rise to quasielastic broadening and an increase in the IFWS. As the quasielastic broadening increases beyond the energy integration range in the IFWS, the IFWS intensity starts to decrease. This results in a peak in the IFWS, where the temperature where the maximum intensity occurs and the broadness of the peak relate to the activation energy and the trial frequency of the dynamics.

For BA_2_PbI_4_, we found that the data can be fitted to a sum of two relaxational processes (Figure 2b,c) with the following activation energies: *E*_a1_ = 67 ± 6 meV and *E*_a2_ = 205 ± 8 meV (upon cooling), and *E*_a1_ = 74 ± 3 meV and *E*_a2_ = 150 ± 7 meV (upon heating). Hence, *E*_a1_ is essentially (within error) the same, whereas *E*_a2_ is different for the cooling and heating runs. By comparing these numbers to reported activation energies for organic cation dynamics in relevant MHPs, we note that -CH_3_ and -NH_3_ rotations in MAPbI_3_ were found to exhibit activation energies of about 50 and 120 meV,^11,9^ respectively. These numbers are quite comparable to the activation energies of ∼70 and ∼150 meV, respectively, observed for BA_2_PbI_4_ in our study and may therefore be tentatively assigned to such dynamics. For the cooling run, the 205 meV activation energy cannot be discriminated into a single process since its contribution lies in both crystal phases, which likely exhibit different relaxational motions with different relaxational times and activation energies. For PEA_2_PbI_4_, we found that the data can be fitted to a single relaxation process with an activation energy of 132 ± 2 meV (see Figure 2d).

We now discuss dynamical structure factors *S*(*q*, ω). Figure 3a shows *S*(*q*, ω) for BA_2_PbI_4_, as measured on IN5. For the LT phase (≤260 K), *S*(*q*, ω) can be fitted to one Lorentzian function, but for the HT phase (≥280 K), two Lorentzian functions are needed. Notably, all of the Lorentzians are characterized by a *q*-independent line width, γ, which suggests that the dynamics are localized in nature. On this basis, we constrained the fitting of *S*(*q*, ω) to a function with globally *q*-independent line widths. Figure 3b shows *S*(*q*, ω) together with the fit for *T* = 350 K and *q* = 1.72 Å^–1^. More details about the fits are given in Experimental Details, and additional fits for different temperatures and *q* values are given in the Supporting Information. The temperature dependence of the corresponding line widths is shown in Figure 3c.

**Figure 3:**
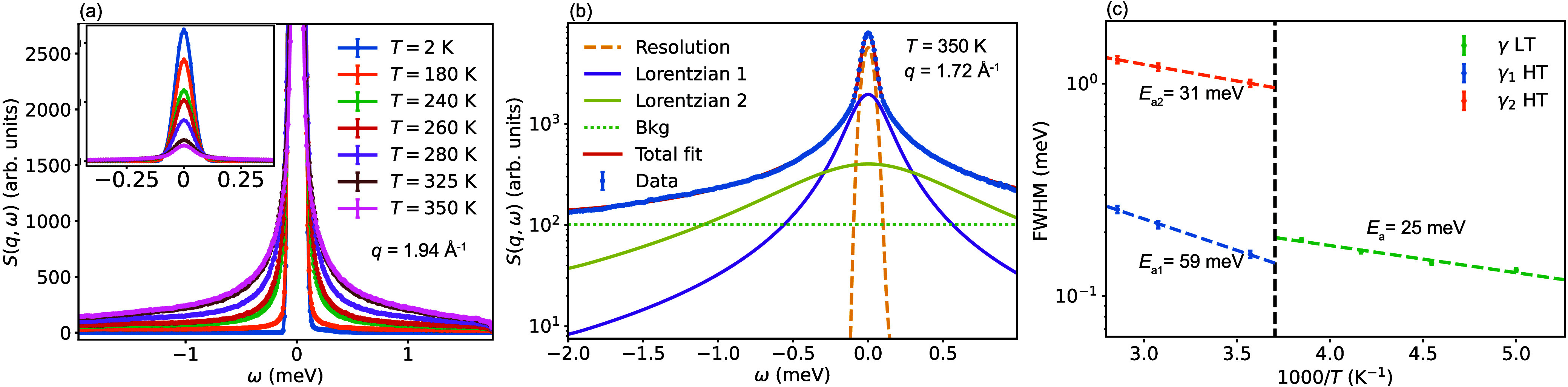
QENS data of BA_2_PbI_4_, as measured on IN5 upon heating. (a) *S*(*q*, ω) for different temperatures at *q* = 1.94 Å^–1^. (b) *S*(*q*, ω) at *q* = 1.72 Å^–1^ in the HT phase at *T* = 350 K, together with the fit to a model with two Lorentzian components. (c) Fitted quasielastic line width (fwhm), measured upon heating. The dashed lines show Arrhenius fits to the data.

The line widths translate into relaxational times in the range of τ = 2ℏ/γ ∼ 1–10 ps and follow Arrhenius behaviors with apparent activation energies of about 25 meV (LT phase) and 59 and 31 meV (HT phase). Thus, these apparent activation energies are lower than what was extracted from the fit of the IFWS (74 and 150 meV (see Figure 2)). This discrepancy may be related to the different time scales probed on BASIS (≈7 – 400 ps) and IN5 (≈0.3 – 13 ps).

Figure 4 compares the elastic incoherent structure factor (EISF) as determined from the experimental data at 200 K (LT phase) and 280 and 350 K (HT phase) compared to calculations based on geometrically feasible jump diffusion models of localized BA cation dynamics from which we can determine the geometry of the dynamics in both phases. In the LT phase (200 K), the EISF data are in good agreement with a model describing 3-fold (*C*_3_) rotations of the -CH_3_ or -NH_3_ group. Note that the EISF models for NH_3_ and CH_3_ rotations would appear almost identical and cannot be separated. However, as the -NH_3_ group is expected to experience stronger hydrogen bonding to neighboring I^–^ anions,^38^ one might speculate this group to be less mobile than the -CH_3_ group. However, it should be clearly pointed out that we cannot discriminate between the CH_3_ and NH_3_ rotations in the fitting to different models.

**Figure 4:**
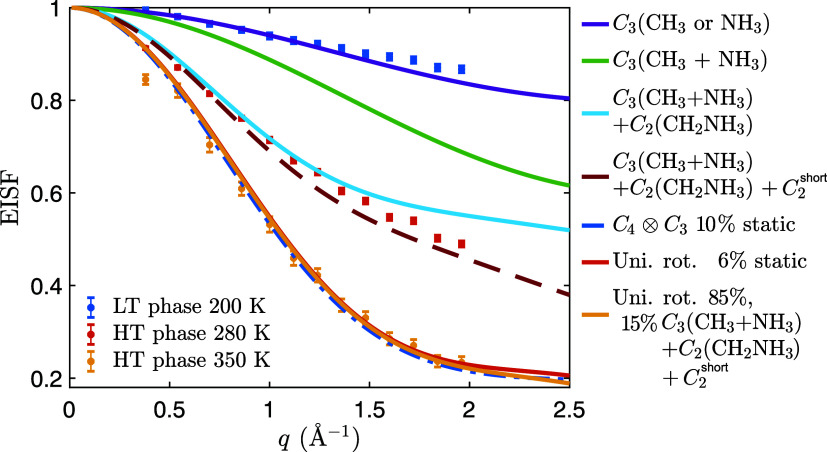
EISF of BA_2_PbI_4_ extracted from the QENS fits on IN5. The data were measured upon heating.

In the HT phase at 280 K, both *C*_3_ rotations of the -NH_3_ and -CH_3_ groups can be expected to be fast enough to be observed on the probed time scale of picoseconds, but a model composed of *C*_3_ rotations of both -NH_3_ and -CH_3_ groups (purple curve) cannot adequately describe the experimental data. Rather, the generally lower value of EISF as compared to that of this model suggests the presence of additional dynamics in the material at this temperature. A good agreement can be found to a model that considers *C*_3_ rotations of both -NH_3_ and -CH_3_ groups, together with *C*_2_ rotation of the -CH_2_NH_3_ group of the BA cation (red dashed curve). This model was used in the previous QENS studies on the similar materials (C_8_H_17_NH_3_)_2_PbI_4_ ^21^ and BA_2_PbBr_4_.^22^ In addition to the -NH_3_ and -CH_3_ groups, together with *C*_2_ rotation of the -CH_2_NH_3_ group, this model takes into account an additional motion of the remaining -CH_2_ groups, which was modeled as a *C*_2_ motion with a small jump distance and is labeled as *C*_2_^short^ in Figure 4. At 350 K, the EISF decreases even further, suggesting that different or additional dynamical processes are taking place on the time scales probed here. For this temperature, we initially considered a model in which the BA cation undergoes uniaxial rotation around its long molecular axis (see Figure 1 for a schematic illustration of these dynamics), but this model predicts a larger fraction of quasielastic scattering than what is experimentally observed. Instead, we find that the 350 K data can be adequately fitted to a model in which only a fraction (85%) of the BA cations performs the isotropic rotation, whereas the remaining fraction (15%) of BA cations undergoes reorientations between preferred orientations using the model used to model the 280 K data (*C*_2_ rotations together with *C*_3_ end-group rotations). One should note that a model that assumes that 6% of the BA cations are static while the remaining performs the uniaxial rotations can also describe the data equally well. This may indicate a dynamical heterogeneity, probably as a result of short-range structural distortions of the material, which is also reflected in the different activation energies obtained from BASIS and IN5. Nevertheless, the results unequivocally show that the whole BA cations (and not only the end groups) are dynamically disordered in the HT phase at ≥300 K with an average relaxation time on the order of 10 ps. However, we should point out that we cannot unequivocally determine whether the BA dynamics are fully continuous (as in the uniaxial rotation model) or have preferred orientations. The latter option could very well be the case, but as the data show more quasielastic scattering than what is predicted from the 2-fold rotational model of the end groups, it must mean that there are more than two sites to these rotations. Note, a recent QENS study has found that the rotational dynamics in the high-temperature phase of BA_2_PbI_4_ can be described in terms of combined *C*_4_ rotations of the whole BA cation around its long molecular axis, combined with *C*_3_ rotations of the end groups.^23^ A comparison of our data to this model suggests that this model can equally well describe our data, under the assumption that 10% of the organic cations are immobile on the probed time scale.

Figure 5a shows the *S*(*q*, ω) of PEA_2_PbI_4_ at *q* = 1.1 Å^–1^ at 20, 250, 300, and 370 K. As one can see, there is clear quasielastic scattering at ≥250 K, which increases in intensity with an increase in temperature. This quasielastic scattering can be described by a single Lorentzian function for all measured temperatures and *q* values (cf. Figure 5b) and with a *q*-independent line width. The line width takes on values in the range of 7–50 μeV for temperatures between 250 and 370 K, which translates into relaxation times (calculated as 2ℏ/fwhm) between 188 and 26 ps. Accordingly, these dynamics are significantly slower than what was observed for BA_2_PbI_4_ in the same temperature range. The corresponding activation energy is approximately 99 meV (Figure 5c), which is similar to the activation energy we have derived from the fitting of the IFWS data. Thus, there is relatively good agreement between these two ways of extracting the activation energy. For comparison, this activation energy is also similar to that calculated for -NH_3_ rotations in MAPbI_3_ ^11^ but higher than that for the combined *C*_3_ rotations found experimentally,^9^ where the -CH_3_ and -NH_3_ groups move together with a lower activation energy.^39^ This indicates that for the BA cation the -NH_3_ and -CH_3_ groups move separately, which gives rise to a higher activation barrier for rotation.

**Figure 5:**
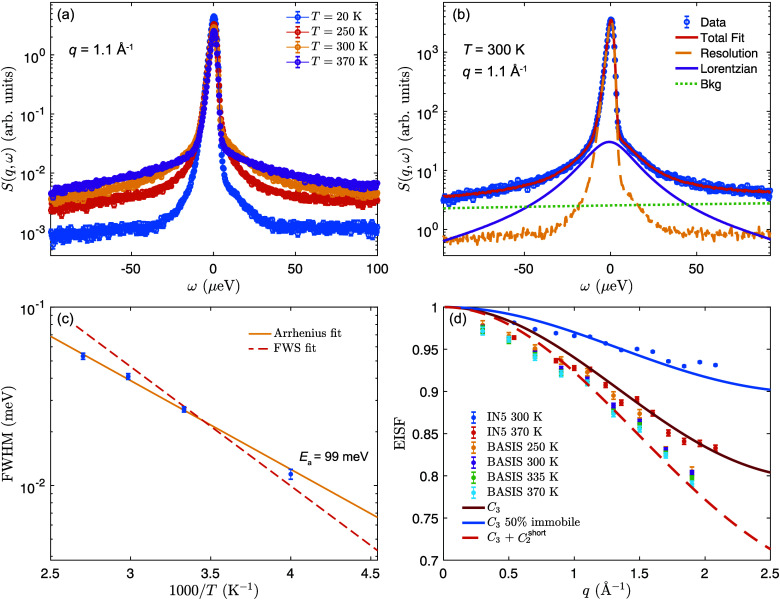
QENS data of PEA_2_PbI_4_. (a) *S*(*q*, ω) for different temperatures at *q* = 1.1 Å^–1^ measured on BASIS. (b) Fit to *S*(*q*, ω) at 300 K. (c) Fitted quasielastic line width as a function of temperature, as measured on BASIS. The solid line shows an Arrhenius fit to the data, and the dashed shows the predicted temperature dependence of the fwhm from the fit to the IFWS data in Figure 2d. (d) EISF of BA_2_PbI_4_ extracted from the QENS fits on BASIS and IN5.

Figure 5d shows the EISF of PEA_2_PbI_4_ at 300 K and at 370 K as obtained from the QENS measurements on IN5 and BASIS. Considering first the IN5 data, at the highest measured temperature (370 K), the EISF can be described well by a model that considers only the *C*_3_ rotations of the -NH_3_ groups. The data at 300 K can be best fitted to the same model but with an immobile fraction of 50%. Considering next the BASIS data, which reflect slightly slower time scale dynamics, we, in contrast, observe a virtually temperature-independent EISF, with slightly lower values than what is predicted for *C*_3_ rotations of the -NH_3_ groups. This could be explained if there is some additional slower relaxational mode present. As the -NH_3_ group rotates, it is likely that the four neighboring hydrogens of the (CH_2_)_2_NH_3_ group also move due to a distortion of the PEA cation. Here, we modeled these additional dynamics as a *C*_2_ model with a small jump distance, similar to that in ref 21. This motion is labeled as *C*_2_^short^ in Figure 5d.

As one can see in the figure, such a model can adequately describe the BASIS data. Therefore, the combined analyses of IN5 and BASIS data suggest that the PEA cation dynamics can be described as *C*_3_ rotations of the -NH_3_ groups together with a slower motion of the -(CH_2_)_2_NH_3_ groups. Note that the phenyl ring part of PEA remains static on the time scales probed here (τ > 0.4 ns) possibly due to the strong intermolecular interactions between the phenyl rings.

By bringing together the results from the QENS data, we can now understand several new features pertaining to organic cation dynamics in BA_2_PbI_4_ and PEA_2_PbI_4_. For BA_2_PbI_4_, measurements upon heating reveal the onset of rotational dynamics at around 100 K, which can be assigned to rotational diffusion of the -CH_3_ and -NH_3_ groups of the BA cations, whereas for temperatures higher than 300 K, we also observe full rotational diffusion of the whole BA cation around its long molecular axis. For PEA_2_PbI_4_, measurements upon heating reveal the onset of rotational dynamics at around 200 K, which can be assigned to rotational diffusion of the -NH_3_ groups of the PEA cations with no sign of additional dynamics at higher temperatures. Therefore, one can conclude that the BA cation dynamics in BA_2_PbI_4_ are spatially more free than the PEA cation dynamics in PEA_2_PbI_4_. In comparison to previously reported QENS studies of layered MHPs, such as (C_8_H_17_NH_3_)_2_PbI_4_,^21^ BA_2_PbBr_4_, ODAPbBr_4_, (GABA)_2_PbBr_4_,^22^ and (BA)_2_(MA)_*n*−1_PbnI_3*n*+1_ (MA = CH_3_NH_3_; *n* = 2 or 3),^40^ the organic cation dynamics in these systems have generally been described as *C*_2_ or *C*_3_ rotations, or combinations thereof, which is markedly different from the much less restricted dynamics of the BA cations in BA_2_PbI_4_, which therefore stands out from the rest.

The rotations of the whole BA cations at ≥300 K, observed here for BA_2_PbI_4_, would mean that there is more dynamical disorder in BA_2_PbI_4_ than in the previously studied materials. This is also expected to influence charge carriers that will experience fluctuations of the potential energy from the organic cation dynamics on a time scale of picoseconds. In MHPs, the photoexcited charge carriers are typically discussed in terms of excitons^41^ or polarons,^42^ which form on a time scale of picoseconds in these materials. In this context, our QENS results are in agreement with a recent computational study, which predicted full rotation of the whole organic cation at room temperature in BA_2_PbBr_4_.^24^

The organic cation dynamics in PEA_2_PbI_4_ are significantly slower than what we observe for BA_2_PbI_4_ at 300 K but similar to what was observed by Koegel et al.^22^ for ODAPbBr_4_ and (GABA)_2_PbBr_4_. We further found that the phenyl ring of PEA remains static on the time scales probed here using QENS, which is in agreement with NMR results showing that the phenyl ring rotations occur on time scales of 10–100 μs.^17^ This would imply that dynamical fluctuations in PEA-based layered perovskites are much more limited due to the stronger intermolecular forces between the PEA cations.

At this point, it is interesting to compare our results on the organic cation dynamics to the materials’ optoelectronic properties. Importantly, BA_2_PbI_4_ and PEA_2_PbI_4_ exhibit some characteristic differences in their optoelectronic properties. In particular, PEA systems (PEA_2_PbX_4_, where X = I and Br) have slower electron–hole recombination and longer carrier lifetimes,^30–32^ and more than 1 order of magnitude faster diffusion and longer exciton diffusion lengths.^33^ The longer lifetimes and diffusion lengths of charge carriers in PEA_2_PbI_4_ may thus be related to its more restricted dynamics, probably as a consequence of stronger intermolecular interactions between PEA cations in this material. We hypothesize that these stronger intermolecular interactions lead to a stronger structural rigidity of the organic substructure, which leads to less scattering of excitons and charge carriers, thus making their lifetimes and diffusion lengths longer.

Finally, we comment on the possible interactions between organic cation dynamics and octahedral tilting dynamics of the PbI_6_ framework, which has been discussed extensively in the literature on MHPs.^43–45^ For PEA_2_PbI_4_ and BA_2_PbI_4_, both materials exhibit octahedral distortions at room temperature, for which BA_2_PbI_4_ exhibits the largest out-of-plane distortion. Both of these materials have been shown to exhibit a heavily damped vibrational dynamics, similar to those of MAPbI_3_,^28^ indicating that a significant organic cation dynamics is not crucial for having heavily damped dynamics of the inorganic substructure. On the other hand, in both BA_2_PbI_4_ and MAPbI_3_,^28^ there is a clear correlation of the onset of organic cation dynamics with a large damping of the phonon modes. This indicates that organic cation dynamics can further broaden the phonon dynamics through large dynamic local distortions.

To conclude, our QENS experiments unravel contrasting organic cation dynamics in the layered lead iodide perovskites BA_2_PbI_4_ and PEA_2_PbI_4_. In PEA_2_PbI_4_, the organic cation dynamics are very restricted in nature. They are limited to rotations of the -NH_3_ groups, which occur on a time scale of about 0.1 ns above 200 K. For BA_2_PbI_4_, the organic cation dynamics are more pronounced and correlated with the structural phase transitions of the material. More specifically, they evolve from *C*_3_ rotations of the -NH_3_ and -CH_3_ groups in the low-temperature phase to rotations of the whole BA cation along the long molecular axis in the high-temperature phase, which becomes more and more free with an increase in temperature in this phase. We hypothesize that the more dynamic organic cations in BA_2_PbI_4_ lead to an additional scattering source for excitons and charge carriers, leading to a reduced lifetime and limited diffusion lengths.

## Experimental Details

*Sample Synthesis and Characterization*. Powder samples of BA_2_PbI_4_ and PEA_2_PbI_4_, approximately 2 g per composition, were synthesized according to reported procedures.^46,47^ Rietveld analysis of room-temperature powder X-ray diffraction patterns shows that both samples are single-phase and in agreement with reported crystal structures^28,29^ (see the Supporting Information).

*Quasielastic Neutron Scattering*. The QENS experiments were performed on the time-of-flight spectrometer IN5 ^48^ at the Institut Laue-Langevin (ILL), Grenoble, France, and on the backscattering spectrometer BASIS^49^ at the Spallation Neutron Source (SNS), Oak Ridge National Laboratory, Oak Ridge, TN. The powder samples were held inside standard cylindrical aluminum sample holders for all measurements. After general and instrument-specific data reductions, which are briefly outlined below, the obtained quantity in each experiment is the measured dynamical structure factor, *S*(*q*, ω), where *q* and ℏω are the wavevector and energy transfer, respectively. The complementarity in using both IN5 and BASIS is that they allow different parts of (*q*, ω) space to be probed with different energy resolutions, meaning that, compared to measuring on only one instrument, information about the dynamics on a larger range of time and length scales can be obtained.

The measurements on IN5 were performed using an incident neutron wavelength of 5 Å, allowing us to probe dynamics featuring relaxation times between ∼0.1 and 15 ps. An energy resolution (at full width at half-maximum (fwhm)) of 0.1 meV and an accessible *q* range of ∼0.2–2 Å^–1^ at the elastic line were obtained. Measurements were taken at temperatures between 2 and 350 K, where the respective 2 K data were used as a resolution function in the fitting of the quasielastic line shape. The fitting of the QENS data was done over the energy interval [−2,1] meV. The measuring time, at each temperature, was approximately 1 h. Data reduction, which included background subtraction, and correction for the energy-dependent efficiency of the detectors were applied to the time-of-flight data with LAMP.^50^

The measurements on BASIS were carried out using Si(111) and Si(311) analyzer crystals. The use of the Si(111) analyzer crystal allowed the probing of dynamics with an energy resolution (at fwhm) and accessible energy transfer range of 3.5 μeV and ±100 μeV, respectively, and the accessible *q* range was ∼0.2–2 Å^–1^ at the elastic line. The use of the Si(311) analyzer crystal allowed probing of dynamics with an energy resolution and accessible energy transfer range of 15 μeV and ±660 μeV, respectively, and the accessible *q* range was ∼0.4–3.8 Å^–1^ at the elastic line. By bringing together the data from the two different setups, it was possible to probe dynamics with relaxation times between ∼2 and 400 ps. Measurements were performed between 20 and 370 K, where the data at the lowest temperature (20 K) were used as the resolution function in the fitting of the quasielastic line shape. The measuring time, for each temperature, was approximately 1.667 h (1 × 10^13^ proton counts on the spallation target) and 2.5 h (1.5 × 10^13^ proton counts on the spallation target) for the Si(111) and Si(311) setups, respectively. Additionally, short (≈10 min, 1 × 10^12^ proton counts on the spallation target) measurements were performed in steps of 10 K on cooling (for both samples) and heating (only for BA_2_PbI_4_) over the temperature range from 2 to 300 K to study the temperature dependence of the elastic and inelastic intensities, which are known as elastic and inelastic fixed window scans (EFWS and IFWSs), respectively. For the EFWS, the elastic intensity was integrated between −3.5 and 3.5 μeV. For the IFWS, the inelastic intensity was integrated between 3.5 and 10 μeV. Data reductions were done within Mantid.^51^ The background from the empty Al sample holder was not subtracted.

*S*(*q*, ω), as measured on both instruments, was fitted to the following function:

$$S\left(q,\omega \right)\propto \left[A_{0}\left(q\right)\delta \left(\omega \right)+\overset{N}{\underset{i}{\sum }}A_{i}\left(q\right)L\left(\omega ;\gamma_{i}\right)\right]⊗R\left(q,\omega \right)+\mathrm{bkg}\left(q\right)$$ 1

where *R*(*q*, ω) is the resolution function of the instrument, $L\left(\omega ;\gamma_{i}\right)$ are Lorentzian functions with fwhm γ_*i*_, bkg­(*q*) is a flat background that is allowed to depend on *q*, and *A*_0_(*q*) and *A*_*i*_(*q*) are the elastic and quasielastic incoherent structure factors, respectively, which were fitted with the constraint that *A*_0_ + ∑_*i*_ *A*_*i*_(*q*) = 1. In the LT phase of BA_2_PbI_4_, and for PEA_2_PbI_4_ at all measured temperatures, one (*N* = 1) Lorentzian function was needed to describe the QENS signal, whereas in the HT phase of BA_2_PbI_4_, two (*N* = 2) Lorentzian functions were needed. Information about the spatial geometry of the dynamics was obtained by the analysis of the elastic incoherent structure factor (EISF), which was calculated with the equation EISF = *A*_0_(*q*)/[*A*_0_ + ∑_*i*_ *A*_*i*_(*q*)].

## Supplementary Material



## Data Availability

Access to the neutron scattering data measured at the ILL is provided in ref 52.

## Notes

Powder X-ray diffraction data with Rietveld analysis of the BA_2_PbI_4_ and PEA_2_PbI_4_ samples, description of the EISF models, and additional fits to the QENS spectra (PDF)
