# Supplementary material for: Organic Cation Dynamics in the Layered Lead Iodide Perovskites BA2PbI4 and PEA2PbI4
Source: J Phys Chem Lett. 2025 Sep 26;16(40):10282–90. doi: 10.1021/acs.jpclett.5c02091 (PMC12516723; doi:10.1021/acs.jpclett.5c02091)
Supplement: Supplementary file 1 [file jz5c02091_si_001.pdf]

Supporting Information:
Organic Cation Dynamics in the Layered Lead Iodide Perovskites BA_2PbI_4 and PEA_2PbI_4

Rasmus Lavén[a], Michael M. Koza[b], Niina H. Jalarvo[c], Marco Moroni[d], Lorenzo Malavasi[d], Maths Karlsson[a,*]

[a]Department of Chemistry and Chemical Engineering, Chalmers University of Technology, Göteborg 41296, Sweden
[b]Institut Laue-Langevin, 71 avenue des Martyrs, Grenoble 38042, France
[c]Chemical and Engineering Materials Division, Oak Ridge National Laboratory, Oak Ridge, Tennessee 37831-6475, U.S.
[d]Department of Chemistry and INSTM, University of Pavia, Viale Taramelli 16, Pavia 27100, Italy.
*Email: maths.karlsson@chalmers.se

S1 Powder X-ray diffraction

The powder X-ray diffraction pattern (Cu Kα radiation) of the samples $\mathrm{PEA_2PbI_4}$ and $\mathrm{BA_2PbI_4}$ are shown in Fig. S1. Both samples are single phase and the diffraction patterns are in accordance with reported crystal structures.[S1,S2] The Rietveld refinements are based on reported structures in the $C2/m$ space group for $\mathrm{PEA_2PbI_4}$[S1], and in the $Pbca$ space group for $\mathrm{BA_2PbI_4}$.[S2]

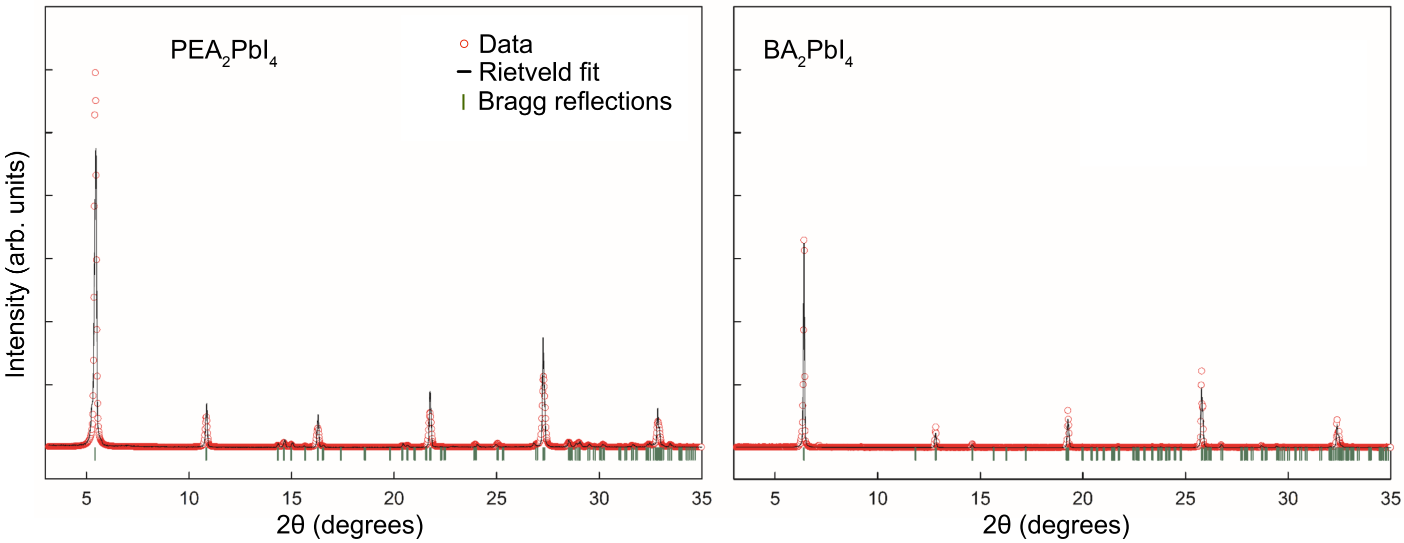

Figure S1 X-ray diffraction data for $\mathrm{PEA_2PbI_4}$ and $\mathrm{BA_2PbI_4}$ together with Rietveld refinements.

S2 EISF models

The C_2 rotation is described by the following EISF

$$\mathrm{EISF}_{C_2} = \frac{1}{2}\left[1 + j_0(qd)\right], \tag{S1}$$

where d is the jump distance, and $j_0(x) = \sin(x)/x$ is the zeroth order spherical Bessel function. To describe the C_2 rotations of the $(CH_2)NH_3$ group of BA, we used the C_2 EISF model with a jump distance of $d = 3$ Å. To describe the quasi librations of the remaining CH_2 groups, we used the C_2 EISF model with a jump distance of $d = 0.9$ Å. The C_3 rotation is described by the following EISF

$$\mathrm{EISF}_{C_3} = \frac{1}{3}\left[1 + 2j_0(qd)\right]. \tag{S2}$$

To describe the C_3 rotations of the NH_3 and CH_3 groups, we used the C_3 EISF model with a jump distance of $d = 1.56$ Å for CH_3 and $d = 1.45$ Å for NH_3.[S3] The uniaxial rotation is described by the following EISF

$$\text{EISF}_{\text{uniaxial}} = \frac{1}{N}\sum_{n=1}^{N} j_0(2rq\sin[n\pi/N]), \tag{S3}$$

where r is the radius of rotation, and we used $r = 1.61$ Å to describe the uniaxial rotation of BA. Note, the 2 CH_2 groups closest to the NH_3 are expected to move on a smaller radius, and we therefore used $r = 0.95$ Å for those H atoms. To approximate a continuous rotation, we used a large value of $N = 300$. An immobile fraction of H atoms was taken into account by the following equation

$$\text{EISF}_{\text{uniaxial, immobile fraction}} = p + (1-p)\frac{1}{N}\sum_{n=1}^{N} j_0(2rq\sin[n\pi/N]), \tag{S4}$$

where p is the fraction of immobile H atoms.

S3 Fits to QENS spectra

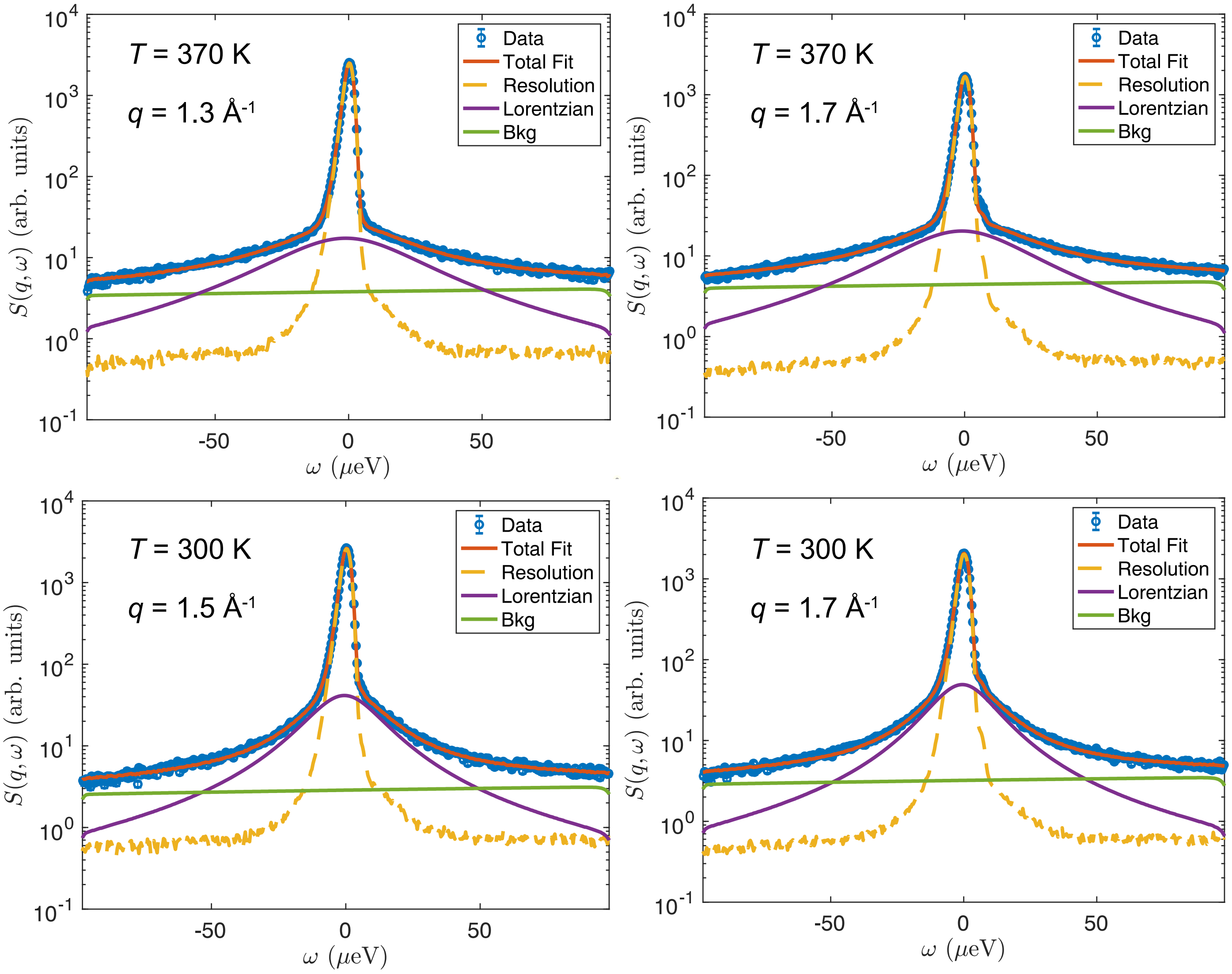

Figure S2 Fits to QENS spectra of PEA_2PbI_4 measured at BASIS.

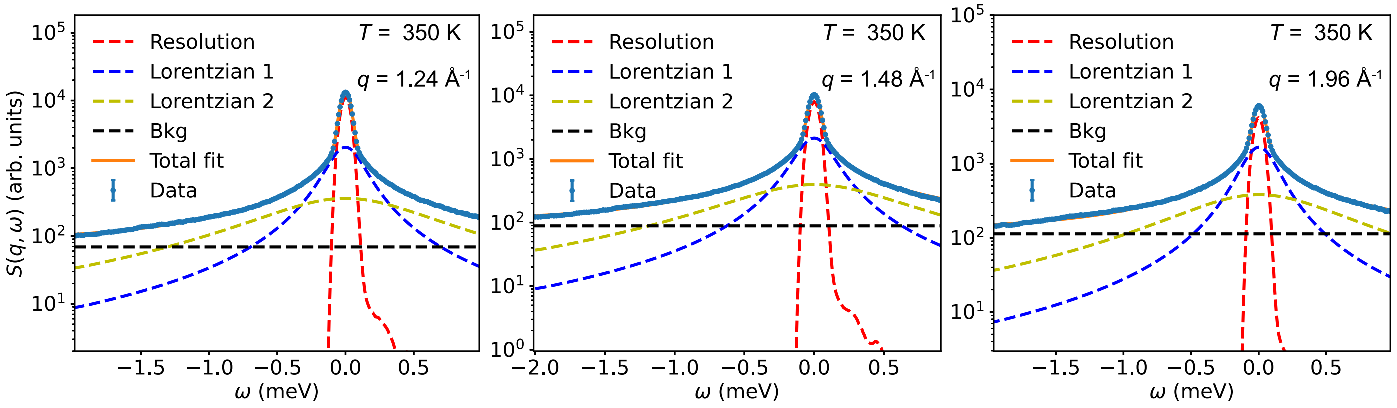

Figure S3 Fits to QENS spectra of BA_2PbI_4 measured at IN5.

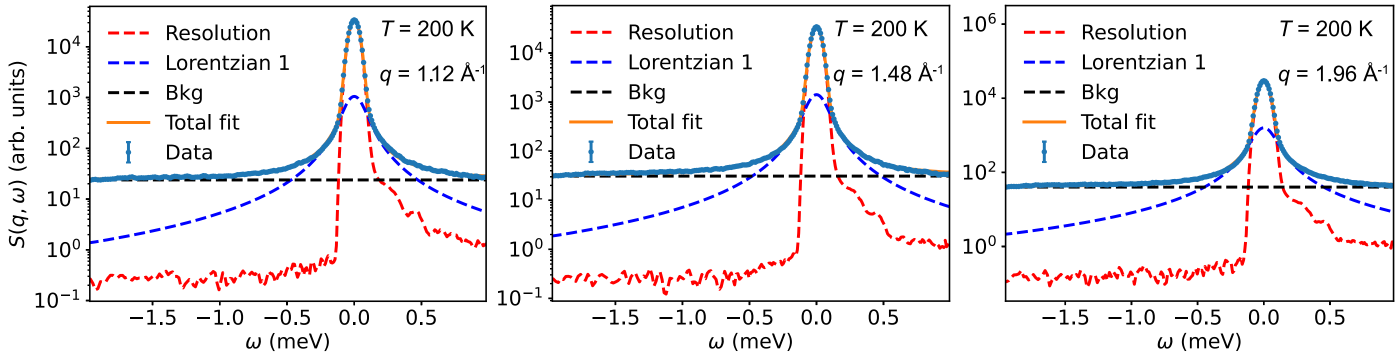

Figure S4 Fits to QENS spectra of BA_2PbI_4 measured at IN5.

References

S1. Calabrese, J.; Jones, N. L.; Harlow, R. L.; Herron, N.; Thorn, D. L.; Wang, Y. Preparation and Characterization of Layered Lead Halide Compounds. *J. Am. Chem. Soc.* **1991**, *113*, 2328–2330.

S2. Billing, D. G.; Lemmerer, A. Synthesis, characterization and phase transitions in the inorganic-organic layered perovskite-type hybrids $[(C_nH_{2n+1}NH_3)_2PbI_4]$, $n = 4$, 5 and 6. *Acta Crystallogr. Sect. B Struct. Sci.* **2007**, *63*, 735–747.

S3. Menahem, M.; Dai, Z.; Aharon, S.; Sharma, R.; Asher, M.; Diskin-Posner, Y.; Korobko, R.; Rappe, A. M.; Yaffe, O. Strongly Anharmonic Octahedral Tilting in Two-Dimensional Hybrid Halide Perovskites. *ACS Nano* **2021**, *15*, 10153–10162.
